# Analytical validation of monoclonal antibody-based ELISA methods for OxPL-apoB and OxPL-apo(a)

**DOI:** 10.1016/j.jlr.2026.100976

**Published:** 2026-01-07

**Authors:** Santica Marcovina, Spenser Smith, Joyce Kornel, Xiaohong Yang, Sotirios Tsimikas

**Affiliations:** 1 Medpace Reference Laboratories, Cincinnati, OH; 2 Vascular Medicine Program, Division of Cardiology, University of California San Diego, La Jolla, CA

**Keywords:** oxidation, lipoproteins, lipoprotein(a), oxidative stress, cardiovascular, therapies

## Abstract

Oxidized phospholipids (OxPL) are bioactive lipid species that circulate bound to apolipoprotein B-100 [apoB] and apolipoprotein(a) [apo(a)] and have been widely studied as biomarkers of oxidative lipid burden. When bound to apolipoprotein B-100 [OxPL-apoB] and apolipoprotein(a) [OxPL-apo(a)], they serve as informative biomarkers for CVD risk prediction, risk reclassification, and therapeutic monitoring, particularly in studies involving RNA-targeted therapies against lipoprotein(a). To date, measurement of OxPL-apoB and OxPL-apo(a) has been limited to research-use assays performed in an academic laboratory without formal clinical laboratory validation. Here we report the first full CLIA-compliant analytical validation of chemiluminescent ELISA methods for OxPL-apoB and OxPL-apo(a), enabling their implementation in a regulated clinical reference laboratory setting. The OxPL-apoB ELISA employs murine monoclonal IgG antibody MB47 to capture apoB-100–containing lipoproteins, while the OxPL-apo(a) employs murine monoclonal IgG antibody LPA4 to capture apo(a)-containing particles. In both assays, OxPL is detected by murine monoclonal IgM antibody biotin-E06. The concentration of OxPL is determined against a standard curve of phosphocholine (PC) equivalents using PC-modified bovine serum albumin. The analytical measuring range of both assays is 1.48–148.48 nmol/L PC-OxPL. Serum and plasma matrices showed minimal bias and were analytically equivalent. In healthy donors, OxPL-apoB levels ranged from <1.48 to 25.23 nmol/L PC-OxPL (mean 4.18, median 1.79 nmol/L), while OxPL-apo(a) levels ranged from <1.48 to 126.94 nmol/L PC-OxPL (mean 31.04, median 6.90 nmol/L), with strong correlation to Lp(a) concentrations (*R*^2^ = 0.82). These assays provide robust tools for quantifying proatherogenic and pro-inflammatory OxPL-lipoprotein complexes in clinical, translational, and pharmacological research settings.

Oxidized phospholipids (OxPL) are bioactive molecules generated during lipid peroxidation and play a central role in the pathogenesis of atherosclerosis (1). Among circulating lipoproteins, OxPL are preferentially bound to apolipoprotein B-100 (apoB)–containing particles, especially lipoprotein(a) [Lp(a)], which carries a disproportionately high OxPL burden due to its unique structural and physiological features (2, 3). OxPL-apoB and OxPL-apo(a) have been extensively evaluated as research biomarkers in epidemiologic and interventional studies, where they have shown consistent associations with cardiovascular outcomes. However, these studies have relied on research-grade assays without formal analytical validation in a CLIA-certified laboratory environment (4, 5, 6, 7, 8, 9, 10, 11).

OxPL-apoB reflects the cumulative number of apoB-containing particles enriched with OxPL and serves as a surrogate marker of systemic oxidative lipid injury (12, 13, 14, 15). Moreover, OxPL-apoB levels strongly correlate with Lp(a) concentrations, reinforcing the pathophysiologic link between these entities (2, 3). Interventional studies show that therapies targeting Lp(a) also potently reduce OxPL-apoB, suggesting its potential as a modifiable biomarker of cardiovascular risk (16, 17, 18, 19, 20). Thus, OxPL-apoB represents both a biomarker and a mechanistic contributor to cardiovascular disease, offering translational utility in risk stratification and therapeutic targeting.

Lp(a) is a genetically inherited lipoprotein particle that plays a central role in atherothrombosis and calcific aortic valve disease (21). It consists of a low-density lipoprotein (LDL)-like core and apolipoprotein(a) [apo(a)], covalently linked to apoB-100. OxPL bind covalently to specific lysine-binding domains on apo(a), making Lp(a) the dominant carrier of circulating OxPL in human plasma. OxPL-apo(a) levels are elevated in individuals with high Lp(a) and are independent predictors of progression to symptomatic heart failure, cardiovascular death, peripheral artery disease, and myocardial infarction (10, 22, 23, 24, 25, 26, 27). Inflammatory modifiers like IL-6 can further amplify OxPL-associated risk in secondary prevention settings (28, 29). As a biomarker reflecting the pathological oxidation burden of Lp(a), OxPL-apo(a) may provide an informative tool to evaluate emerging Lp(a)-lowering therapies.

To support the adoption of OxPL-apoB and OxPL-apo(a) as biomarkers in pharmacological and clinical research, analytically validated and standardized assays are essential. Such assays must demonstrate accuracy, precision, reproducibility, and matrix equivalence to ensure reliable quantification across applications. The objective of this study was therefore to perform a full CLIA-compliant analytical validation of chemiluminescent ELISA methods for OxPL-apoB and OxPL-apo(a) in human serum and EDTA plasma.

## Materials and Methods

### Antibodies

Both the OxPL-apoB and the OxPL-apo(a) ELISA assays utilize a two-antibody system consisting of a capture antibody specific to the lipoprotein of interest and a detection antibody that targets OxPL. For the OxPL-apoB assay, the capture antibody is recombinant murine monoclonal IgG MB47, which binds an epitope on human apolipoprotein B-100 (30), ensuring selective binding to all apoB-containing particles. For the OxPL-apo(a) assay, the capture antibody is recombinant murine monoclonal IgG antibody LPA4 (31), which recognizes the 14-amino acid peptide TRNYCRNPDAEIRP present on apo(a) KIV_5_, KIV_7_, and KIV_8_ and the partial sequence NYCRNPDA present on KIV_2_, enabling specific capture of Lp(a). In both assays, OxPL are detected using the well-characterized biotinylated murine IgM monoclonal antibody E06 (1, 32), which binds to phosphocholine-containing oxidized but not native phospholipids, thereby ensuring specificity for oxidatively modified phospholipids.

### Antibody-based ELISA for OxPL-apoB and OxPL-apo(a) quantification

The assays were replicated at the Medpace Reference Laboratories based on the predicate methods developed at the University of California, San Diego (UCSD) (1). MB47, LPA4, and E06 monoclonal antibodies were produced under controlled conditions from hybridoma-derived and recombinant expression systems (GenScript). Lot-to-lot comparability and functional equivalence were verified prior to assay implementation using standardized quality-control materials within the CLIA laboratory.

**Fig. 1:**
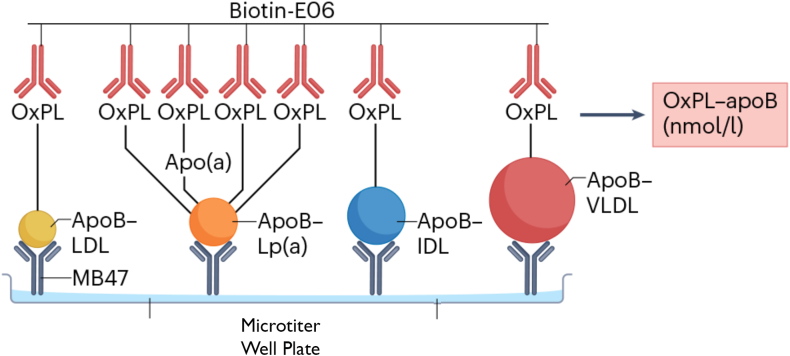
To quantify OxPL-apoB, the MB47 antibody is plated overnight at 5 μg/ml to bind apoB-100 on the microtiter plate; the excess material is then washed off, and the plasma sample is added at a 1:50 dilution to allow apoB-100 to bind to the immobilized MB47. Because MB47 recognizes all apoB-100-containing particles with similar specificity, the apoB-100 captured on the plate will reflect the proportion of apoB-100-containing particles that are present in the plasma sample. For example, if Lp(a) levels are elevated, a higher proportion of apoB-100 from Lp(a) will be captured on the microtiter well plate. Biotin-modified E06 (1 μg/ml) is then added to the plate to bind to the OxPLs present on apoB-100-containing lipoproteins, and streptavidin modified with alkaline phosphatase is added to bind to the biotin–E06 in a 1:1 ratio. A chemiluminescent substrate for alkaline phosphatase (Lumi-Phos) is then added to generate light, which is directly proportional to the amount of phosphocholine-containing OxPLs present in the microtiter well plate and reported as RLU emitted per 100 ms. RLUs are then converted to nanomoles per liter using a standard curve of phosphocholine (PC) equivalents. (1).

**Fig. 2:**
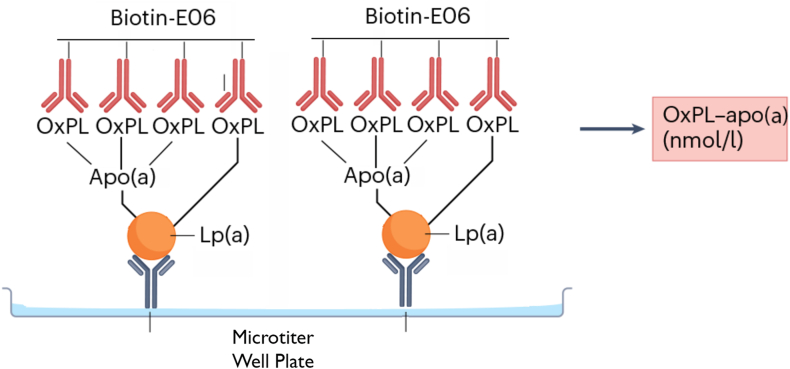
To quantify OxPL-apo(a), the LPA4 antibody is plated overnight at 5 μg/ml to bind apo(a) on the microtiter plate; the excess material is then washed off, and the plasma sample is added at a 1:50 dilution to allow Lp(a)/apo(a) to bind to the immobilized LPA4. Biotin-modified E06 (1 μg/ml) is then added to the plate to bind to the OxPLs present on Lp(a)/apo(a)-containing lipoproteins, and streptavidin modified with alkaline phosphatase is added to bind to the biotin–E06 in a 1:1 ratio. A chemiluminescent substrate for alkaline phosphatase (Lumi-Phos) is then added to generate light, which is directly proportional to the amount of PC-containing OxPLs present in the microtiter well plate and reported as RLU emitted per 100 ms. RLUs are then converted to nanomoles per liter using a standard curve of PC equivalents.

For both the OxPL-apoB and the OxPL-apo(a) chemiluminescent ELISAs, 96-well microtiter plates (Eppendorf) were coated overnight at 4°C with 40 μl per well of either MB47 (5 μg/ml for OxPL-apoB) or LPA4 (5 μg/ml for OxPL-apo(a)) in Dulbecco’s phosphate-buffered saline (pH 7.0). Plates were then blocked with 1% bovine serum albumin (BSA; Sigma-Aldrich) in Tris-buffered saline (TBS) for 1 h at room temperature. Cartoons of the methodologies for the OxPL-apoB and OxPL-apo(a) assays are shown in Figures 1 and 2, respectively.

Serum or EDTA plasma samples are diluted 1:50 for OxPL-apoB and OxPL-apo(a) in TBS containing 0.025 mM Butylated hydroxytoluene (BHT), 1% BSA, 0.27 mM EDTA, and 0.02% sodium azide. Samples are added in duplicate (40 μl/well) and incubated for 1 h at room temperature. Following three washes with TBS-EDTA, biotinylated E06 (1 μg/ml) is added to each well and incubated for an additional hour.

After washing, NeutrAvidin-conjugated alkaline phosphatase (AP; Thermo Fisher Scientific) with activators (1 M MgCl_2_, 0.1 mM ZnCl_2_) is applied for 1 h, followed by detection using 25 μl/well of Lumi-Phos 530 chemiluminescent substrate (Lumigen Inc). Plates are read using a BioTek Synergy H1 plate reader (Agilent Technologies), and relative light units per 100 ms (RLU) are recorded. Assay calibration curves are generated using serial dilutions of a pooled high-OxPL serum reference material (Medpace Reference Laboratories) whose values are referenced to known quantities of PC-BSA, and results are interpolated using a 4-parameter logistic fit.

### Validation standards and performance criteria

The OxPL-apoB and OxPL-apo(a) assays were implemented at the Medpace Reference Laboratories and the analytical validation was performed in accordance with the Clinical Laboratory Improvement Amendments (CLIA) and College of American Pathologists (CAP) accreditation requirements. Testing procedures were based on the Clinical and Laboratory Standard Institute (CLSI) method evaluation standard. Acceptability criteria for quantitation limits, as well as storage and freeze/thaw stability, were based on the 2022 FDA M10 Bioanalytical Method Validation and Study Samples (FDA M10 BMVSS) guidance. Validation parameters included within-run precision, total imprecision, linearity, analytical measuring range (AMR), lower and upper limits of quantification (LLOQ, ULOQ), spike and recovery, dilutability, accuracy, matrix equivalency, and reference ranges in normolipidemic individuals.

### Sample handling

Serum and EDTA plasma from healthy donors were collected under standard conditions. Plasma was processed within 15 min of collection, while serum was allowed to clot for ≥30 min. Samples were centrifuged at 1800–2200 × g, aliquoted, and frozen prior to analysis.

### Repeatability (within-run precision) and reproducibility (total imprecision)

To evaluate the repeatability of the two assays, four Quality Controls and seven samples from individual donors were analyzed in 12 replicates in a single run. All replicates were prepared and analyzed under identical assay conditions to evaluate within-run imprecision, with performance expressed as the coefficient of variation (CV). An acceptance threshold of ≤10% CV was used to determine satisfactory repeatability.

For evaluation of the reproducibility or total imprecision of the two assays, the same Quality Controls utilized for the repeatability, were analyzed in duplicate at the beginning and end of every plate for six days. A different set of seven samples from individual donors were analyzed in duplicate at the beginning and at the end of every plate for seven days. An acceptance threshold of ≤20% was used to determine satisfactory reproducibility.

### Analytical measuring range and linearity

The analytical measuring range and linearity for both the OxPL-apoB and OxPL-apo(a) assays were established using a seven-point serial dilution of a high OxPL serum reference material calibrated to phosphocholine-oxidized phospholipid (PC-OxPL) concentrations. Dilution levels ranged from 0.0297 to 2.970 nmol/L PC-OxPL on the assay plate, corresponding to 1.48–148.48 nmol/L PC-OxPL in undiluted sample equivalents, based on the sample dilution protocols of 1:50 for OxPL-apoB and OxPL-apo(a). Each concentration level was tested in duplicate across six independent assay runs. Accuracy was assessed by percent recovery relative to expected values, and precision by calculating CV at each dilution. The measured concentration at each level should be within ±15.0% of the expected mean with a % CV ≤15.0%, except at the limits of quantitation (LOQ), where it should be within 25.0%. Linearity was further evaluated using linear and second-order polynomial regression models, with acceptable performance defined as percent recovery within ±20% and CVs ≤25% across all levels.

### Limit of quantitation

The lower and upper limits of quantitation (LLOQ and ULOQ) for both assays were defined based on the lowest and highest concentrations in the validated analytical measuring range that met predefined acceptance criteria. Criteria included a coefficient of variation (CV) ≤25% and percent recovery within ±25% of the expected value. The limits were established using duplicate testing of a seven-point serial dilution series from a pooled high-OxPL serum reference standard. Dilutions were tested over six independent runs, and signal response, precision, and accuracy were evaluated at each concentration.

### Assay accuracy—spike and recovery

Spike and recovery experiments were conducted to evaluate assay accuracy and matrix compatibility. For both the OxPL-apoB and the OxPL-apo(a) assays, five unique plasma samples with low endogenous OxPL levels were selected and spiked with a high-OxPL serum pool (Medpace Reference Laboratories) at a defined volume-to-volume ratio. Spiked and unspiked samples were assayed in duplicate. Recovery was calculated as the observed increase in OxPL concentration divided by the expected increase based on the amount of spike added. Acceptable recovery was predefined as 80–120%, with bias (observed vs. expected) within ±20%.

### Dilutability

Dilutability was assessed by testing pooled plasma samples with high endogenous OxPL levels at standard dilution (1:50 for OxPL-apoB and OxPL-apo(a)) and at three additional serial dilutions (1:25, 1:75, and 1:200 for OxPL-apoB and OxPL-apo(a)). Each dilution level was measured in duplicate. Back-calculated concentrations at the higher dilutions were adjusted for the dilution factor and compared to the concentration at the standard dilution. Percent recovery and bias were calculated to evaluate assay performance. Acceptable dilutability was defined as recovery between 80–120% and absolute percent bias ≤20%.

### Matrix equivalency

Matrix equivalency was evaluated by comparing paired serum and EDTA plasma samples from forty healthy donors for each assay. Samples were processed in parallel and diluted according to validated protocols: 1:50 for the OxPL-apoB and OxPL-apo(a) assay. Each pair was analyzed in duplicate, and the relative percent bias between the matched serum and plasma samples was calculated. A bias threshold of ±15% was predefined as the acceptance criterion for matrix equivalency.

### Stability assessment

Stability of the OxPL-apoB and OxPL-apo(a) assays was evaluated under various conditions, including preanalytical handling, long-term storage, and freeze/thaw stress. For preanalytical stability, six donor samples were stored at room temperature (18–25°C) or refrigerated (2–8°C) and tested after 2, 4, and 24 h.

Long-term stability was assessed by storing five sample aliquots at −20°C or −70°C and testing them over 12 months. Freeze/thaw stability was examined after three cycles in samples stored either at −70°C or at −20°C. Bias was calculated relative to baseline, with acceptance defined as ≤±20% in at least 4 of the 5 samples per time point.

### Expected values in healthy normolipidemic donors

To determine expected values for each assay, human EDTA plasma and serum samples from healthy normolipidemic donors were analyzed using the validated OxPL-apoB and OxPL-apo(a) ELISAs. Samples were processed according to standardized pre-analytical protocols, including centrifugation within 15–30 min of collection and freezing at −70°C. Each specimen was tested in duplicate, and OxPL concentrations were quantified using the plate-specific calibration curves. Data were summarized using descriptive statistics, including range, mean, and median. For the OxPL-apo(a) assay, correlation with Lp(a) molar concentrations measured in the same samples was also assessed.

### Statistical analyses

Data were analyzed using Analyse-it (v5.68), and acceptability criteria were based on FDA 2022 M10 BMVSS Guidance and Westgard Total Allowable Error (TaE) thresholds. The mean values of the assay comparison were analyzed using Deming Regression and Bland Altman (33) plots to determine bias. Linearity was determined to evaluate the statistical significance of second and third-order polynomials in the fit between the expected, theoretical concentration, and the observed concentration.

## Results

### Precision

#### Repeatability (within-run imprecision) and reproducibility (total imprecision)

##### OxPL-apoB

For OxPL-apoB, the repeatability (% CV) for the four quality control samples and for the seven samples from individual donors was within the established criteria of 10%, ranging from 5.6% to 9.6% (Supplemental Table S1) and from 4.7% to 9.7%, respectively (Supplemental Table S2).

For the reproducibility or total imprecision, the %CV for the four Quality Controls and for the seven samples from individual donors was within the established criteria of 20%, ranging from 14.5% to 19.7% (Supplemental Table S3) and from 15.8% to 20.0%, respectively (Supplemental Table S4).

##### OxPL-apo(a)

For OxPL-apo(a), the repeatability (% CV) for the four quality control samples and the seven samples from individual donors was within acceptance criteria (≤10.0%), with CV%, ranging from 5.9% to 9.7% (Supplemental Table S5) and 3.8–8.2%, respectively (Supplemental Table S6).

For the reproducibility or total imprecision, the %CV for the four quality controls and for the seven samples from individual donors was within the established criteria of 20%, ranging from 5.5% to 17.7% (Supplemental Table S7) and 15.0% to 19.8%, respectively (Supplemental Table S8).

#### Analytical measuring range (AMR)

**Fig. 3:**
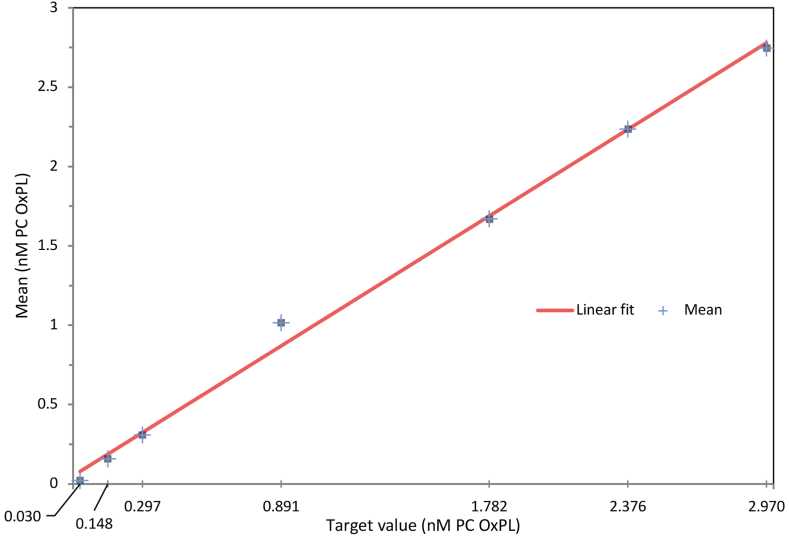
The standard curve was generated by diluting PC-BSA-(2) powder with 10 ml DPBS to make a 1 mg/ml stock as described in the method protocol. The 1 mg/ml stock was diluted ×20 to make a 50 μg/ml stock. Seven calibrators spanning the AMR were prepared according to the method protocol and run in duplicate eight times on five different days.

Both the OxPL-apoB and OxPL-apo(a) ELISAs demonstrated acceptable performance for calibrators diluted in sample dilution buffer over a linear range from 0.0297 to 2.970 nmol/L PC-OxPL (Fig. 3). Accounting for the minimum required dilution of ×50, the analytical measuring range for serum or EDTA plasma is 1.48 nmol/L PC-OxPL to 148.48 nmol/L PC-OxPL.

#### Lower limit of quantitation for OxPL-apoB and OxPL-apo(a)

Per the FDA 2022 M10 BMVSS Guidance, at the LLOQ, the AMR should not deviate from the actual or predicated value by more than 25.0%, and total imprecision should not be greater than 25.0%. For OxPL-apoB and OxPL-apo(a), the lowest standard solution that demonstrated acceptable performance in the sample dilution buffer at the LLOQ was 0.0297 nmol/L PC-OxPL (Level 1), with an observed % bias and % CV (reproducibility) of −24.2% and 41.6%, respectively. The level 1 calibrator did not meet acceptance criteria for the % CV; however, since the values are so low and the absolute bias was −0.007, it was determined that the variation within this level of calibrator is acceptable. The assay’s LLOQ was 0.0297 nmol/L PC-OxPL; accounting for the required 50-fold dilution, the functional LLOQ is 1.48 nmol/L PC-OxPL.

#### Upper limit of quantitation for OxPL-apoB and OxPL-apo(a)

The highest standard solution that demonstrated acceptable performance in the sample dilution buffer at the ULOQ was 2.97 nmol/L PC-OxPL (Level 7), with an observed % bias and % CV (reproducibility) of −7.5% and 9.4%, respectively. The assay’s ULOQ was 1.48 nmol/L PC-OxPL. Considering the minimum required ×50 dilution, the functional ULOQ is 148.48 nmol/L PC-OxPL.

#### Spike and recovery for OxPL-apoB and OxPL-apo(a)

The spike and recovery assessment demonstrated acceptable accuracy of the OxPL-apoB assay in EDTA plasma. Five donor samples were spiked with the High Precision Control at two levels (1:10 and 1:20 ratios), and all ten conditions met the predefined acceptance criterion of ±20% bias. The observed percent bias ranged from −19.6% to +6.5%, with corresponding recoveries between 80.4% and 106.5% (Supplemental Table S9).

The spike and recovery evaluation demonstrated acceptable accuracy of the OxPL-apo(a) assay across a range of concentrations. Five donor samples were spiked with the High Precision Control at two levels (1:10 and 1:20 ratios), and all ten conditions met the predefined acceptance criterion of ±20% bias. The observed percent bias ranged from −8.5% to 13.1%, with corresponding recoveries between 91.5% and 113.4% (Supplemental Table S10).

#### Dilutability for OxPL-apoB and OxPL-apo(a)

The concentrations of OxPL-apoB and OxPL-apo(a) in three EDTA plasma samples were analyzed before and after diluting ×25, ×50 (baseline), ×75, and ×200 with sample dilution buffer. Each was run in duplicate, and the mean, SD, % CV, bias, and % bias were calculated. The measured concentration of the diluted sample should be within ±20.0% of the baseline concentration after correcting for the dilution.

For OxPL-apoB all three samples, dilutions from ×25 through ×200 failed to meet the acceptance criteria, with the % bias ranging from −58.3% to 103.5%. Therefore, OxPL-apoB samples above the AMR cannot be diluted outside of the ×50 assay protocol recommended dilution.

For OxPL-apo(a), all three dilution points did not meet the predefined accuracy criterion of ±20% bias, with recoveries ranging from −93.0% to 178.8%. For all three samples, dilutability failed to meet the acceptance criteria from ×25 through ×200 dilution. Therefore, OxPL-apo(a) samples above the AMR cannot be diluted outside of the ×50 assay protocol recommended dilution.

#### Matrix equivalency OxPL-apoB and OxPL-apo(a)

For the OxPL-apoB assay, forty EDTA plasma and serum samples collected from the same individual were analyzed in parallel for OxPL-apoB. EDTA plasma and serum results are considered equivalent if bias is within ±15%. Bland-Altman analysis identified a mean % bias of −2.43% between the two matrices (Supplemental Fig. S1). Deming Regression estimated the slope as 0.9538 and the intercept as 0.01684, with a correlation coefficient of 0.957 (Supplemental Fig. S2).

For the OxPL-apo(a) assay, Bland-Altman analysis identified a mean % bias of 1.01% between the two matrices (Supplemental Fig. S3). Deming Regression estimated the slope as 1.016 and the intercept as 0.03711, with a correlation coefficient of 0.995. Results are presented in the tables and graphs below (Supplemental Fig. S4).

#### Range of expected OxPL-apoB and OxPL-apo(a) values in healthy normolipidemic donors

A range of expected values was established by analyzing 40 EDTA plasma samples from presumably healthy volunteers. The OxPL-apoB values in EDTA plasma collected from healthy volunteers ranged from <1.48 nmol/L to 25.23 nmol/L PC-OxPL, with a mean of 4.18 nmol/L and median 1.79 nmol/L PC-OxPL (Table 1).

**Table 1:** Expected values for OxPL-apoB and OxPL-apo(a) in 40 healthy normolipidemic subjects

	OxPL-apoB	OxPL-apo(a)
Number	40	40
Mean	4.18	31.04
Median	1.79	6.92
Std. Deviation	5.11	40.33
Minimum	<1.48	<1.48
Maximum	25.23	126.94
Percentiles		
25	1.48	1.48
50	1.79	6.92
75	3.76	68.10
80	4.74	77.02
90	12.66	105.49
95	18.08	116.99

For OxPL-apo(a) values ranged from <1.48 nmol/L to 126.94 nmol/L PC-OxPL, with a mean of 31.04 nmol/L and median 6.92 nmol/L PC-OxPL (Table 1). For this group, Lp(a) molar concentrations, measured by Roche Tina-quant Gen.2 kit on a Roche c502 analyzer, were available, and a strong positive correlation was observed between OxPL-apo(a) and total Lp(a) molar concentration (*R*^2^ = 0.82), consistent with previous findings that Lp(a) is the major carrier of OxPL in human plasma (1).

**Fig. 4:**
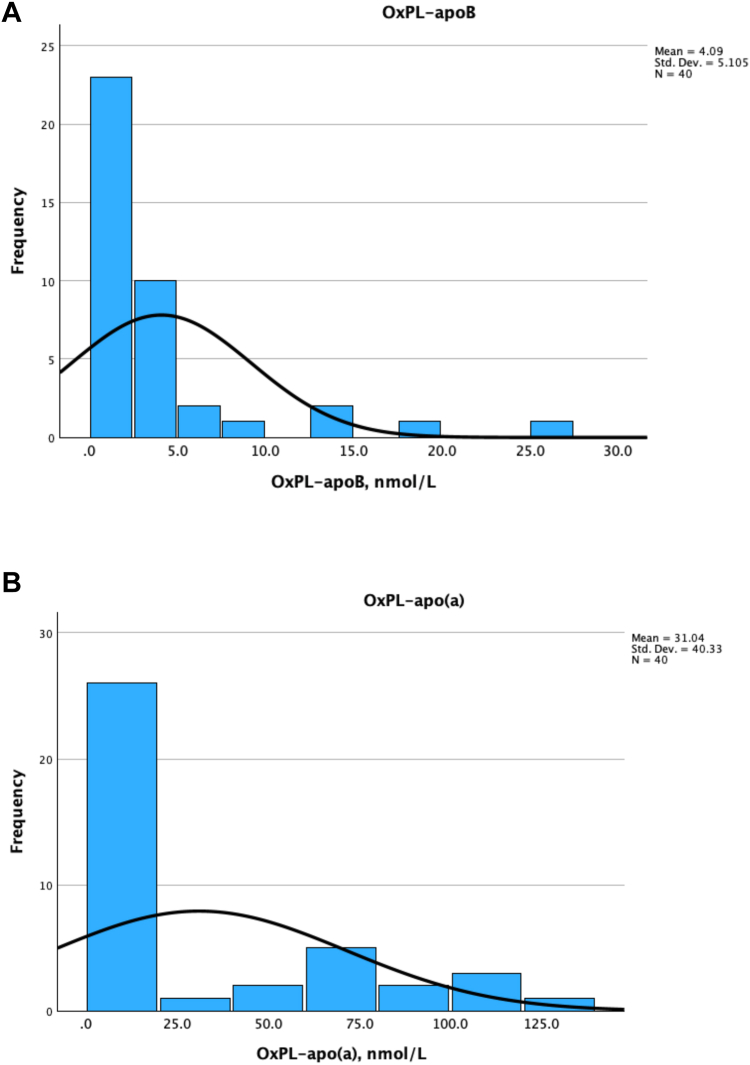
Frequency distribution of OxPL-apoB and OxPL-apo(a) levels in healthy normolipidemic individuals. A: OxPL-apoB. B: OxPL-apo(a).

As shown previously for both OxPL-apoB and OxPL-apo(a) (1), results were not normally distributed, and normality could not be achieved through log or Box-Cox Power transformations (Fig. 4).

Because serum and EDTA plasma expected values ranges are similar, which is supported by the matrix comparison data, the same expected values range will be used for both matrices.

### Stability assessment

The stability assessment of the OxPL-apoB and OxPL-apo(a) assays demonstrated acceptable sample stability under defined storage conditions. EDTA plasma samples are stable at room temperature (18–25°C) for up to 4 h, after which substantial variability is observed; therefore, freezing at −70°C within 1 h is recommended to minimize ex vivo oxidation. Samples stored at 2–8°C remained stable for up to 24 h. Long-term storage at −20°C preserved stability for up to 6 months, but significant degradation was observed by 9 months and failure by 12 months. In contrast, samples stored at −70°C remained stable for at least 12 months, with all measured values within acceptable limits. Therefore, stability at −70°C will continue to be regularly monitored. Freeze–thaw stability was limited to a single cycle for −20°C samples, whereas −70°C samples tolerated up to two cycles without compromising assay performance.

## Discussion

This study reports the first comprehensive CLIA-compliant analytical validation of chemiluminescent ELISA assays for OxPL-apoB and OxPL-apo(a), enabling their transition from academic research assays to regulated clinical laboratory use. Both assays employ recombinant monoclonal antibodies, MB47 for apoB and LPA4 for apo(a), to ensure high specificity and batch-to-batch consistency, with biotin-E06 IgM serving as the detection antibody targeting PC-containing OxPL. The assays demonstrated strong analytical performance, including linearity across a wide dynamic range (1.48–148.48 nmol/L PC-OxPL), good within- and between-run precision, matrix equivalency between serum and plasma, and accurate spike and recovery characteristics. Expected range of values in healthy normolipidemic individuals is also provided. These results support the use of both OxPL-apoB and OxPL-apo(a) assays for clinical and research purposes, especially in studies exploring CVD risk stratification and therapeutic responses with PCSK9i and Lp(a) lowering agents.

OxPL-apoB has been extensively studied as a biomarker reflecting the oxidative burden on circulating apoB-containing lipoproteins (1). In association studies, elevated levels have been linked to endothelial dysfunction, anatomical diseases including coronary, carotid artery disease and peripheral artery disease, prediction of first and subsequent myocardial infarction and stroke, heart failure, and calcific aortic stenosis, independent of traditional lipid metrics (4, 5, 6, 7, 8, 9, 10, 11). In interventional studies, statins tend to mildly raise OxPL-apoB as they also raise its main lipoprotein carrier Lp(a) (34, 35, 36, 37, 38, 39, 40, 41). Niacin (40) and PCSK9 inhibitors such as evolocumab (42) and alirocumab (43) modestly reduce OxPL-apoB levels. Importantly, in the ODYSSEY Outcomes trial, in patients with recent ACS receiving optimized statin treatment, elevated OxPL-apoB levels predicted MACE, a relationship abrogated by alirocumab. The interaction of OxPL-apoB and Lp(a) in the placebo group indicated that OxPL-apoB independently predicts MACE when Lp(a) levels are relatively low (43). OxPL-apoB levels are potently (∼60-70%) acutely reduced following apheresis, particularly in patients with baseline elevated Lp(a) (44). However, the most potent effect on OxPL-apoB has been noted with the antisense oligonucleotide targeting *LPA* mRNA, pelacarsen (88%), (16, 17, 18) short interfering RNA olpasiran (>90%) (19) and the oral Lp(a) disruptor muvalaplin (60–70%) (20).

OxPL-apo(a), while highly correlated with Lp(a) levels, provides a more specific reflection of the atherogenic potential of Lp(a) due to the covalent attachment of OxPL to the apo(a) moiety. Published studies have demonstrated that OxPL-apo(a) levels are elevated in patients with myocardial infarction, peripheral arterial disease, heart failure, and calcific aortic valve disease (10, 22, 23, 24, 25, 26, 27, 39). In the phase 2 trial of pelacarsen, OxPL-apo(a) levels declined in parallel with Lp(a), demonstrating dose responsiveness and providing mechanistic insight into therapeutic effects (18). Unlike Lp(a) total mass or molar concentration, OxPL-apo(a) may better reflect the inflammatory and pro-calcific properties of Lp(a), especially in contexts that may reflect the benefit of anti-inflammatory agents such as colchicine (28, 29).

Compared to OxPL-apoB, the absolute values of OxPL-apo(a) have consistently been higher across all studies to date (1). This difference arises from both methodological and pathophysiological factors. Methodologically, the OxPL-apoB assay uses the MB47 antibody to capture all apoB-containing lipoproteins proportionally to their plasma concentrations. Since Lp(a) is a minor component among apoB particles, even at high concentrations (45), its contribution to the total assay signal is limited. In contrast, OxPL-apo(a) assays specifically isolate Lp(a) particles via the apo(a) component, thereby enriching for OxPL content. From a pathophysiological standpoint, Lp(a) is the primary lipoprotein carrier of OxPL in human plasma. The apo(a) component of Lp(a) contains specific lysine-binding domains in the KIV_10_ domain that covalently bind OxPL to the protein backbone. Additionally, OxPL are non-covalently retained within the lipid phase of Lp(a) particles (2, 3). While OxPL-apoB reflects the total OxPL on all apoB-100–containing particles, mainly LDL and, to a lesser extent, Lp(a), most circulating OxPLs are selectively enriched on Lp(a). In contrast, OxPL associated with LDL are more transient, non-covalently bound, and present at lower concentrations. Moreover, each Lp(a) particle likely carries a higher molar density of OxPL than LDL due to its unique structure and affinity for OxPL. Consequently, assays targeting apo(a) detect a concentrated OxPL signal, leading to consistently higher OxPL-apo(a) values than those observed in OxPL-apoB assays. These differences underscore not only the analytical specificity of the assays but also the distinct pathophysiological role of OxPL-rich Lp(a) particles in atherogenesis.

Mass spectrometry–based approaches quantify individual oxidized phospholipid species with high chemical specificity but typically require lipid extraction and do not preserve native lipoprotein context (46). In contrast, the OxPL-apoB and OxPL-apo(a) ELISAs quantify phosphocholine-containing OxPL epitopes directly on intact apoB- and apo(a)-containing particles. These approaches are therefore complementary rather than redundant, with the ELISAs uniquely suited for high-throughput clinical trial applications where preservation of lipoprotein context, scalability, and standardization are essential.

OxPL-apoB and OxPL-apo(a) assays may be particularly valuable in therapeutic studies aimed at lowering Lp(a), such as antisense oligonucleotides (e.g., pelacarsen) and small interfering RNAs (e.g., olpasiran, lepodisiran, zerlasiran), assembly inhibitors (e.g., muvalaplin) and *LPA* gene editing approaches. As these agents reduce Lp(a) levels by over 80%, concurrent measurement of OxPL-apoB and OxPL-apo(a) can help determine whether reductions in Lp(a) translate into decreased oxidative burden and downstream inflammation. These assays could serve as intermediate biomarkers to assess biological plausibility and mechanism of action before hard cardiovascular outcomes are available. Moreover, they offer broader utility in therapies that affect multiple apoB-containing lipoproteins or modulate the redox state of plasma lipids.

The validated assays are implemented as laboratory-developed tests within a CLIA-certified reference laboratory and are currently available for use in translational research and clinical trials, including studies evaluating emerging Lp(a)-lowering therapies. Their analytical performance characteristics support longitudinal monitoring and multicenter deployment.

In conclusion, these analytically validated OxPL-apoB and OxPL-apo(a) ELISAs provide robust, standardized tools suitable for regulated clinical laboratory use. Their validation addresses a critical barrier to widespread adoption and supports their application in clinical trials and translational research settings.

## Data Availability

Data that support the plots within this publication and other findings of this study are available from the corresponding authors upon request.

## Supplemental Data

This article contains supplemental data.

## Conflict of Interest

The authors declare the following financial interests/personal relationships which may be considered as potential competing interests: S. M. M., S. S. and J. K. are employees of Medpace Reference Laboratories. S. M. M. reports consulting roles for Denka. S. T. is a co-inventor and receives royalties on patents held by the University of California on monoclonal antibodies directed to Lp(a) and oxidized phospholipids, is a co-founder and has an equity interest in Oxitope, Kleanthi Diagnostics, and Megaron, and has a dual appointment at UCSD and Ionis Pharmaceuticals. All other authors declare that they have no conflicts of interest with the contents of this article.

## Supplemental data

Method Supplement
