# Supplementary material for: Analytical validation of monoclonal antibody-based ELISA methods for OxPL-apoB and OxPL-apo(a)
Source: J Lipid Res. 2026 Jan 7;67(2):100976. doi: 10.1016/j.jlr.2026.100976 (PMC12857368; doi:10.1016/j.jlr.2026.100976)
Supplement: Method Supplement [file mmc1.docx]

# Supplement

**Analytical Validation of Monoclonal Antibody-Based ELISA Methods for OxPL-apoB and OxPL-apo(a)**

# Santica Marcovina^1^, Spenser Smith^1^, Joyce Kornel^1^,

# Xiaohong Yang^2^, Sotirios Tsimikas^2^

# ^1^Medpace Reference Laboratories, Cincinnati, Ohio, USA ^2^Vascular Medicine Program, Division of Cardiology, University of California San Diego, La Jolla, California, USA

**Table S1:** Summary of OxPL-apoB QC Repeatability.

| Replicate # | High QC | High Precision | Med Precision Control | Hi/B Pool |
| --- | --- | --- | --- | --- |
| 1 | 49.86 | 14.67 | 8.13 | 31.35 |
| 2 | 46.96 | 15.99 | 9.41 | 34.07 |
| 3 | 48.93 | 13.93 | 8.99 | 30.40 |
| 4 | 44.04 | 14.95 | 8.35 | 33.20 |
| 5 | 47.54 | 15.28 | 8.01 | 32.01 |
| 6 | 44.53 | 16.72 | 8.05 | 31.94 |
| 7 | 44.13 | 16.15 | 8.80 | 33.97 |
| 8 | 44.93 | 15.78 | 8.31 | 34.26 |
| 9 | 51.70 | 11.27 | 9.48 | 32.53 |
| 10 | 47.84 | 15.60 | 8.60 | 36.10 |
| 11 | 51.17 | 16.33 | 8.01 | 34.40 |
| 12 | 48.01 | 15.57 | 8.04 | 36.57 |
|  |  |  |  |  |
| Mean (nmol/L PC-OxPL) | 47.47 | 15.19 | 8.51 | 33.40 |
| SD (nmol/L PC-OxPL) | 2.66 | 1.45 | 0.54 | 1.86 |
| CV (%) | **5.6%** | **9.6%** | **6.3%** | **5.6%** |

**Table S2:** Summary of OxPL-apoB Sample Repeatability.

| Replicate # | 1 | 2 | 3 | 4 | 5 | 6 | 7 |
| --- | --- | --- | --- | --- | --- | --- | --- |
| 1 | 67.06 | 54.09 | 59.57 | 60.26 | 56.27 | 59.97 | 46.05 |
| 2 | 70.09 | 56.34 | 56.90 | 55.29 | 58.46 | 61.54 | 43.37 |
| 3 | 71.70 | 62.15 | 60.11 | 61.85 | 69.46 | 73.15 | 41.55 |
| 4 | 71.67 | 52.68 | 58.92 | 62.14 | 65.96 | 62.58 | 43.54 |
| 5 | 69.10 | 59.36 | 54.66 | 62.71 | 62.05 | 71.30 | 45.28 |
| 6 | 57.50 | 58.41 | 54.42 | 61.09 | 59.85 | 75.22 | 42.29 |
| 7 | 63.38 | 63.20 | 60.79 | 61.21 | 56.94 | 58.47 | 41.36 |
| 8 | 64.96 | 49.55 | 59.86 | 66.45 | 68.32 | 59.98 | 41.24 |
| 9 | 60.53 | 55.07 | 61.43 | 63.31 | 63.01 | 61.76 | 48.09 |
| 10 | 65.21 | 52.06 | 56.23 | 72.98 | 64.80 | 61.69 | 49.63 |
| 11 | 62.61 | 53.32 | 53.12 | 62.11 | 65.35 | 55.79 | 42.59 |
| 12 | 62.11 | 48.46 | 57.65 | 65.04 | 60.14 | 61.12 | 43.96 |
|  |  |  |  |  |  |  |  |
| Mean (nmol/L PC-OxPL) | 65.49 | 55.39 | 57.80 | 62.87 | 62.55 | 63.55 | 44.08 |
| SD (nmol/L PC-OxPL) | 4.54 | 4.66 | 2.74 | 4.18 | 4.34 | 6.16 | 2.70 |
| CV (%) | **6.9%** | **8.4%** | **4.7%** | **6.7%** | **6.9%** | **9.7%** | **6.1%** |

**Table 3:** Summary of OxPL-apoB QC Reproducibility.

| Run |  | High QC | High Precision | Med Precision Control | Hi/B Pool |
| --- | --- | --- | --- | --- | --- |
| 1 |  | 37.21 | 13.50 | 14.94 | 41.68 |
| 1 |  | 37.18 | 13.54 | 13.41 | 38.08 |
| 1 |  | 48.79 | 14.06 | 11.16 | 35.30 |
| 1 |  | 57.84 | 17.96 | 9.04 | 38.47 |
| 2 |  | 69.04 | 19.93 | 12.14 | 31.16 |
| 2 |  | 74.61 | 15.42 | 11.18 | 36.76 |
| 2 |  | 61.58 | 17.53 | 10.90 | 38.18 |
| 2 |  | 69.31 | 19.33 | 13.23 | 36.48 |
| 3 |  | 48.64 | 18.30 | 11.05 | 39.28 |
| 3 |  | 63.29 | 21.46 | 10.09 | 40.11 |
| 3 |  | 52.10 | 22.80 | 11.76 | 38.77 |
| 3 |  | 62.28 | 16.75 | 11.26 | 40.20 |
| 4 |  | 47.10 | 17.66 | 12.44 | 36.19 |
| 4 |  | 54.78 | outlier | 12.64 | 42.83 |
| 4 |  | 59.19 | 21.51 | 11.86 | 32.16 |
| 4 |  | 47.81 | 16.60 | 11.62 | 36.87 |
| 5 |  | 56.92 | 21.38 | 10.99 | 37.79 |
| 5 |  | 49.82 | 25.47 | 11.36 | 34.46 |
| 5 |  | 54.43 | 25.94 | 11.95 | 38.59 |
| 5 |  | 33.63 | 19.37 | 11.06 | 39.89 |
| 6 |  | 50.18 | 20.27 | 10.05 | 37.21 |
| 6 |  | 56.13 | 20.38 | 9.41 | 31.35 |
| 6 |  | 50.31 | 20.73 | 8.99 | 40.97 |
| 6 |  | 55.08 | 22.57 | 9.78 | 34.07 |
| 7 |  | 51.98 | 22.10 | outlier | 34.22 |
| 7 |  | 41.06 | 14.70 | 7.05 | 29.64 |
| 7 |  | 41.28 | 18.63 | 8.32 | 31.65 |
| 7 |  | 44.31 | 14.51 | 7.62 | 30.62 |
| 8 |  | 44.94 | 14.75 | 10.60 | 27.63 |
| 8 |  | 49.02 | 15.32 | 7.81 | 30.39 |
| 8 |  | 48.48 | 21.09 | 9.18 | 34.52 |
| 8 |  | 34.98 | 21.30 | 10.33 | 36.26 |
| 9 |  | 38.40 | 15.05 | 10.93 | 48.59 |
| 9 |  | 38.62 | 20.58 | 9.61 | 43.44 |
| 9 |  | 41.65 | 12.03 | 12.45 | 46.99 |
| 9 |  | 42.28 | 15.96 | 6.94 | 35.32 |
| 10 |  | 46.36 | 12.85 | 13.23 | 44.15 |
| 10 |  | 45.81 | 12.66 | 11.63 | 38.69 |
| 10 |  | 38.54 | 18.43 | 8.29 | 33.67 |
| 10 |  | 58.80 | 18.64 | 7.26 | 41.40 |
| Mean (nmol/L PC- OxPL) | | 50.09 | 18.23 | 10.60 | 37.10 |
| SD (nmol/L PC-OxPL) | | 9.87 | 3.55 | 1.91 | 3.10 |
| CV (%) | | **19.7%** | **19.4%** | **18.4%** | **14.5%** |
|  |  |  |  |  |  |

**Table S4:** Summary of OxPL-apoB Sample Reproducibility.

| Run | | 1 | | 2 | | 3 | | 4 | | 5 | | 6 | | 7 | |  |  |
| --- | --- | --- | --- | --- | --- | --- | --- | --- | --- | --- | --- | --- | --- | --- | --- | --- | --- |
| 1  1  1  1  2  2  2  2  3  3  3  3  4  4  4  4  5  5  5  5  6  6  6  6  7  7  7  7 | | 66.63 | | 55.26 | | 40.15 | | 39.85 | | 50.48 | | 44.84 | | 46.11 | | |  |
|  |  | 68.07 | | 57.90 | | 43.59 | | 38.99 | | 50.73 | | 33.50 | | 44.25 | | |  |
|  |  | 64.86 | | 54.42 | | 46.66 | | 40.44 | | 54.18 | | 29.00 | | 46.62 | | |  |
|  |  | 68.46 | | 57.24 | | 43.48 | | 36.52 | | 42.72 | | 38.32 | | 45.35 | | |  |
|  |  | 67.52 | | 58.06 | | 50.71 | | 51.83 | | 54.76 | | 54.74 | | 49.63 | | |  |
|  |  | 64.79 | | 61.60 | | 48.79 | | 48.00 | | 62.15 | | 53.19 | | 48.09 | | |  |
|  |  | 69.63 | | 55.96 | | 43.50 | | 48.11 | | 49.87 | | 48.37 | | 41.28 | | |  |
|  |  | 67.16 | | 51.97 | | 43.78 | | 40.88 | | 43.78 | | 45.32 | | 40.98 | | |  |
|  |  | 56.65 | | 48.80 | | 33.63 | | 39.13 | | 44.43 | | 42.08 | | 41.30 | | |  |
|  |  | 55.74 | | 50.45 | | 35.21 | | 35.50 | | 44.01 | | 38.68 | | 36.10 | | |  |
|  |  | 55.88 | | 46.55 | | 35.20 | | 35.24 | | 41.13 | | 37.09 | | 31.49 | | |  |
|  |  | 56.12 | | 42.76 | | 33.46 | | 30.48 | | 39.83 | | 38.34 | | 36.70 | | |  |
|  |  | 65.32 | | 36.98 | | 44.75 | | 41.77 | | 51.55 | | 49.10 | | 46.62 | | |  |
|  |  | 60.64 | | 36.95 | | 45.88 | | 34.98 | | 45.98 | | 46.30 | | 38.00 | | |  |
|  |  | 57.44 | | 35.14 | | 46.22 | | 45.51 | | 45.41 | | 46.47 | | 40.10 | | |  |
|  |  | 58.97 | | 37.44 | | 42.92 | | 39.92 | | 47.93 | | 44.38 | | 46.15 | | |  |
|  |  | 78.66 | | 57.16 | | 51.91 | | 40.95 | | 64.91 | | 44.94 | | 63.82 | | |  |
|  |  | 77.58 | | 67.72 | | 42.03 | | 38.07 | | 65.37 | | 56.76 | | 62.70 | | |  |
|  |  | 84.23 | | 56.54 | | 53.12 | | 51.44 | | 63.56 | | 61.58 | | 58.11 | | |  |
|  |  | 81.71 | | 57.35 | | 51.03 | | 53.34 | | 61.68 | | 47.97 | | 59.70 | | |  |
|  |  | 100.21 | | 50.89 | | 55.37 | | 46.32 | | 50.86 | | 53.56 | | 63.76 | | |  |
|  |  | 98.17 | | 51.64 | | 54.45 | | 48.80 | | 66.36 | | 61.04 | | 66.02 | | |  |
|  |  | 89.86 | | 52.33 | | 59.27 | | 57.41 | | 64.28 | | 63.49 | | 57.50 | | |  |
|  |  | 87.64 | | 46.48 | | 56.86 | | 42.53 | | 65.99 | | 67.08 | | 43.25 | | |  |
|  |  | 89.46 | | 60.63 | | 57.54 | | 56.92 | | 60.37 | | 62.42 | | 58.64 | | |  |
|  |  | 89.20 | | 62.20 | | 56.20 | | 55.31 | | 58.33 | | 54.33 | | 59.12 | | |  |
|  |  | 73.71 | | 63.44 | | 53.26 | | 50.19 | | 60.34 | | 58.90 | | 53.87 | | |  |
|  |  | 76.02 | | 62.36 | | 49.56 | | 51.97 | | 54.89 | | 58.51 | | 53.34 | | |  |
| Mean (nmol/L PC- OxPL) |  | | 72.51 | | 52.72 | | 47.09 | | 44.30 | | 53.78 | | 49.30 | | 49.24 | | |
| SD (nmol/L PC-OxPL) |  | | 13.27 | | 8.73 | | 7.43 | | 7.37 | | 8.58 | | 9.84 | | 9.67 | | |
| CV (%) | | **18.3%** | | **16.6%** | | **15.8%** | | **16.6%** | | **16.0%** | | **20.0%** | | **19.6%** | | |  |

**Table 5:** Summary of OxPL-apo(a) QC Repeatability.

| Replicate # | High QC | High Precision | Med Precision Control | Hi/B Pool |
| --- | --- | --- | --- | --- |
| 1 | 140.73 | 84.19 | 27.82 | 49.17 |
| 2 | 131.59 | 86.84 | 24.66 | 55.02 |
| 3 | 159.68 | 85.50 | 31.58 | 50.61 |
| 4 | 159.04 | 95.56 | 25.46 | 50.22 |
| 5 | 154.33 | 86.89 | 29.30 | 54.36 |
| 6 | 154.64 | 81.60 | 25.12 | 55.15 |
| 7 | 154.57 | 87.09 | 26.55 | 54.16 |
| 8 | 147.84 | 94.95 | 29.76 | 56.95 |
| 9 | 165.19 | 92.80 | 30.73 | 52.54 |
| 10 | 153.94 | 94.80 | 24.89 | 50.59 |
| 11 | 159.71 | 93.94 | 28.96 | 54.53 |
| 12 | 145.65 | 81.94 | 32.25 | 48.93 |
| Mean (nmol/L PC-OxPL) | 152.24 | 88.84 | 28.09 | 52.69 |
| SD (nmol/L PC-OxPL) | 9.37 | 5.25 | 2.73 | 2.69 |
| CV (%) | **6.2%** | **5.9%** | **9.7%** | **5.1%** |

**Table 6:** Summary of OxPL-apo(a) Sample Repeatability.

| Replicate # | 1 | 2 | 3 | 4 | 5 | 6 | 7 |
| --- | --- | --- | --- | --- | --- | --- | --- |
| 1 | 109.88 | 83.43 | 57.06 | 58.95 | 79.52 | 93.92 | 75.54 |
| 2 | 117.21 | 83.43 | 57.74 | 55.57 | 70.52 | 95.49 | 79.75 |
| 3 | 100.72 | 81.23 | 53.01 | 53.82 | 75.67 | 92.93 | 71.49 |
| 4 | 104.74 | 79.20 | 57.87 | 60.20 | 74.01 | 89.72 | 75.32 |
| 5 | 102.23 | 79.01 | 57.90 | 55.75 | 76.28 | 85.63 | 68.47 |
| 6 | 100.94 | 80.03 | 58.11 | 62.97 | 72.68 | 84.29 | 72.99 |
| 7 | 93.87 | 78.86 | 61.70 | 58.91 | 76.90 | 86.94 | 74.56 |
| 8 | 104.15 | 85.69 | 66.47 | 64.43 | 73.19 | 87.37 | 73.49 |
| 9 | 102.12 | 83.71 | 58.76 | 66.53 | 79.40 | 87.43 | 79.52 |
| 10 | 105.36 | 95.35 | 58.28 | 67.60 | 77.52 | 93.17 | 83.43 |
| 11 | 104.90 | 87.68 | 48.45 | 54.61 | 75.87 | 98.29 | 77.00 |
| 12 | 108.52 | 87.54 | 55.36 | 54.43 | 72.01 | 98.11 | 79.73 |
| Mean (nmol/L PC-OxPL) | 104.55 | 83.76 | 57.56 | 59.48 | 75.30 | 91.11 | 75.94 |
| SD (nmol/L PC-OxPL) | 5.71 | 4.82 | 4.35 | 4.90 | 2.86 | 4.85 | 4.17 |
| CV (%) | **5.5%** | **5.8%** | **7.6%** | **8.2%** | **3.8%** | **5.3%** | **5.5%** |

**Table S7.** Summary of OxPL-apo(a) QC Reproducibility.

| Run |  | High QC | High Precision | Med Precision Control | Hi/B Pool |
| --- | --- | --- | --- | --- | --- |
| 1 |  | 111.99 | 76.26 | 31.53 | 49.95 |
| 1 |  | 120.12 | 78.70 | 34.43 | 49.46 |
| 1 |  | 126.16 | 56.28 | 32.07 | 51.82 |
| 1 |  | 122.48 | 61.85 | 33.56 | 48.53 |
| 2 |  | 187.37 | 86.83 | 29.74 | 48.35 |
| 2 |  | 187.86 | 99.48 | 28.46 | 50.81 |
| 2 |  | 166.47 | 90.87 | 31.97 | 48.92 |
| 2 |  | 159.97 | 81.13 | 32.95 | 51.38 |
| 3 |  | 142.17 | 92.40 | 25.85 | 47.11 |
| 3 |  | 142.96 | 111.69 | 25.73 | 49.93 |
| 3 |  | 153.09 | 83.80 | 33.24 | 49.77 |
| 3 |  | 143.47 | 86.54 | 24.36 | 49.84 |
| 4 |  | 131.62 | 64.60 | 28.84 | 46.91 |
| 4 |  | 126.58 | 81.38 | 25.41 | 44.88 |
| 4 |  | 123.67 | 89.59 | 24.77 | 46.98 |
| 4 |  | 122.65 | 82.34 | 28.52 | 47.80 |
| 5 |  | 140.51 | 104.08 | 29.30 | 49.17 |
| 5 |  | 145.10 | 102.73 | 25.12 | 55.02 |
| 5 |  | 149.95 | 77.50 | 26.55 | 50.61 |
| 5 |  | 153.70 | 74.88 | 29.76 | 50.22 |
| 6 |  | 155.72 | 86.98 | 22.32 | 45.93 |
| 6 |  | 139.25 | 88.71 | 25.74 | 50.41 |
| 6 |  | 137.87 | 70.15 | 25.55 | 44.73 |
| 6 |  | 117.62 | 69.67 | 20.94 | 49.15 |
| 7 |  | 131.59 | 86.84 | 22.32 | 49.29 |
| 7 |  | 138.66 | 85.50 | 25.74 | 46.72 |
| 7 |  | 145.65 | 81.60 | 25.55 | 46.67 |
| 7 |  | 140.73 | 81.94 | 20.94 | 48.53 |
| 8 |  | 103.52 | 57.50 | 28.90 | 48.09 |
| 8 |  | 111.26 | 74.57 | 26.53 | 43.50 |
| 8 |  | 107.98 | 80.07 | 27.71 | 48.62 |
| 8 |  | 93.64 | 84.94 | 24.33 | 42.64 |
| 9 |  | 100.41 | 67.87 | 23.54 | 48.60 |
| 9 |  | 101.69 | 63.87 | 25.15 | 48.56 |
| 9 |  | 104.11 | 65.81 | 29.74 | 46.25 |
| 9 |  | 93.95 | 68.59 | 25.21 | 44.23 |
| Mean (nmol/L PC- OxPL) | | 132.82 | 80.49 | 27.29 | 48.32 |
| SD (nmol/L PC-OxPL) | | 23.55 | 12.91 | 3.99 | 2.64 |
| CV (%) | | **17.7%** | **16.0%** | **14.6%** | **5.5%** |

**Table S8.** Summary of OxPL-apo(a) Sample Reproducibility.

| Run |  | 1 | 2 | 3 | 4 | 5 | 6 | 7 |
| --- | --- | --- | --- | --- | --- | --- | --- | --- |
| 1 |  | 87.48 | 68.16 | 65.15 | 62.86 | 70.82 | 71.01 | 86.51 |
| 1 |  | 86.90 | 79.42 | 60.81 | 69.84 | 75.59 | 85.84 | 88.89 |
| 1 |  | 80.14 | 47.65 | 64.55 | 69.81 | 78.38 | 78.72 | 85.00 |
| 1 |  | 90.92 | 66.60 | 61.50 | 76.19 | 82.07 | 81.45 | 73.18 |
| 2 |  | 82.88 | 73.96 | 63.54 | 66.51 | 75.24 | 71.63 | 69.80 |
| 2 |  | 84.32 | 65.19 | 58.27 | 63.21 | 68.84 | 73.21 | 83.71 |
| 2 |  | 78.86 | 66.53 | 60.28 | 60.16 | 64.55 | 57.68 | 61.81 |
| 2 |  | 85.50 | 67.22 | 58.56 | 63.98 | 63.37 | 68.70 | 80.06 |
| 3 |  | 83.14 | 72.08 | 65.60 | 65.27 | 73.71 | 74.67 | 77.58 |
| 3 |  | 85.59 | 71.79 | 59.57 | 64.97 | 71.42 | 77.52 | 98.83 |
| 3 |  | 82.49 | 67.94 | 60.15 | 58.73 | 68.17 | 55.78 | 72.54 |
| 3 |  | 94.68 | 71.40 | 61.52 | 54.07 | 66.24 | 73.27 | 75.72 |
| 4 |  | 89.91 | 73.51 | 74.27 | 68.96 | 70.68 | 84.18 | 81.73 |
| 4 |  | 80.21 | 62.92 | 66.93 | 72.06 | 74.52 | 72.66 | 81.21 |
| 4 |  | 75.75 | 55.12 | 65.18 | 74.51 | 75.01 | 69.09 | 82.30 |
| 4 |  | 75.57 | 60.30 | 67.60 | 69.02 | 67.68 | 67.43 | 82.81 |
| 5 |  | 108.96 | 84.88 | 81.60 | 93.53 | 97.38 | 108.43 | 110.37 |
| 5 |  | 115.80 | 81.37 | 83.37 | 96.17 | 92.05 | 104.58 | 105.11 |
| 5 |  | 105.56 | 77.95 | 91.07 | 88.93 | 88.97 | 92.71 | 108.51 |
| 5 |  | 104.59 | 80.75 | 89.72 | 92.53 | 82.80 | 92.96 | 111.60 |
| 6 |  | 120.04 | 75.41 | 91.33 | 102.43 | 95.77 | 100.42 | 100.42 |
| 6 |  | 117.34 | 82.49 | 94.15 | 96.66 | 99.64 | 111.68 | 111.68 |
| 6 |  | 110.89 | 74.91 | 93.60 | 101.83 | 114.44 | 98.87 | 114.73 |
| 6 |  | 113.37 | 78.10 | 94.33 | 93.96 | 99.78 | 83.08 | 101.48 |
| 7 |  | 112.15 | 100.68 | 96.85 | 98.39 | 104.76 | 105.49 | 119.95 |
| 7 |  | 110.25 | 101.66 | 95.61 | 98.29 | 99.84 | 104.25 | 106.47 |
| 7 |  | 102.66 | 97.89 | 94.41 | 93.58 | 94.78 | 102.87 | 114.16 |
| 7 |  | 102.22 | 102.20 | 95.39 | 86.68 | 90.44 | 100.78 | 107.91 |
| Mean (nmol/L PC- OxPL) | | 95.29 | 75.29 | 75.53 | 78.68 | 82.39 | 84.61 | 92.65 |
| SD (nmol/L PC-OxPL) | | 14.31 | 13.38 | 14.98 | 15.54 | 14.18 | 16.10 | 16.46 |
| CV (%) | | **15.0%** | **17.8%** | **19.8%** | **19.7%** | **17.2%** | **19.0%** | **17.8%** |

**Table S9:** Spike and Recovery Results for OxPL-apoB.

|  |  |  | **Baseline** | | **Spike** | | **Expected** | **Observed** | **Bias** |
| --- | --- | --- | --- | --- | --- | --- | --- | --- | --- |
| **Sample** | **Dilution** | | *Concentration (nmol/L PC-OxPL)* | *Vol. used (µL)* | *Concentration (nmol/L PC-OxPL)* | *Vol. used (µL)* | *Concentration (nmol/L PC-OxPL)* | *Concentration (nmol/L PC-OxPL)* | *(%)* |
| 1 | 1:10 | | 15.61 | 90 | 18.23 | 10 | 15.87 | 14.86 | -6.4% |
|  | 1:20 | | 15.61 | 190 | 18.23 | 10 | 15.74 | 14.11 | -10.4% |
| 2 | 1:10 | | 25.15 | 90 | 18.23 | 10 | 24.46 | 25.81 | 5.5% |
|  | 1:20 | | 25.16 | 190 | 18.23 | 10 | 24.81 | 24.13 | -2.8% |
| 3 | 1:10 | | 19.38 | 90 | 18.23 | 10 | 19.27 | 20.52 | 6.5% |
|  | 1:20 | | 19.38 | 190 | 18.23 | 10 | 19.32 | 20.17 | 4.4% |
| 4 | 1:10 | | 13.78 | 90 | 18.23 | 10 | 14.23 | 11.78 | -17.2% |
|  | 1:20 | | 13.78 | 190 | 18.23 | 10 | 14.00 | 12.29 | -12.2% |
| 5 | 1:10 | | 5.40 | 90 | 18.23 | 10 | 6.68 | 5.40 | -19.2% |
|  | 1:20 | | 5.40 | 190 | 18.23 | 10 | 6.04 | 4.86 | -19.6% |
|  |  | |  |  |  |  |  |  |  |

**Table S10:** Spike and Recovery Results for OxPL-apo(a).

|  |  |  | **Baseline** | | **Spike** | | **Expected** | **Observed** | **Bias** |
| --- | --- | --- | --- | --- | --- | --- | --- | --- | --- |
| **Sample** | **Dilution** | | *Concentration (nmol/L PC-OxPL)* | *Vol. used (µL)* | *Concentration (nmol/L PC-OxPL)* | *Vol. used (µL)* | *Concentration (nmol/L PC-OxPL)* | *Concentration (nmol/L PC-OxPL)* | *(%)* |
| 1 | 1:10 | | 37.46 | 90 | 80.49 | 10 | 42.02 | 47.23 | 13.1% |
|  | 1:20 | | 37.46 | 190 | 80.49 | 10 | 39.74 | 36.23 | -8.5% |
| 2 | 1:10 | | 90.29 | 90 | 80.49 | 10 | 89.57 | 87.27 | -2.3% |
|  | 1:20 | | 90.29 | 190 | 80.49 | 10 | 89.93 | 88.85 | -1.1% |
| 3 | 1:10 | | 79.32 | 90 | 80.49 | 10 | 79.69 | 78.04 | -1.8% |
|  | 1:20 | | 79.32 | 190 | 80.49 | 10 | 79.50 | 83.13 | 4.7% |
| 4 | 1:10 | | 39.97 | 90 | 80.49 | 10 | 44.27 | 45.72 | 3.9% |
|  | 1:20 | | 39.97 | 190 | 80.49 | 10 | 42.12 | 44.03 | 4.9% |
| 5 | 1:10 | | 27.51 | 90 | 80.49 | 10 | 33.06 | 34.37 | 4.8% |
|  | 1:20 | | 27.51 | 190 | 80.49 | 10 | 30.28 | 32.77 | 8.7% |

**Figure S1.** Matrix correlation of EDTA plasma and serum samples for OxPL-apoB assay.

**Figure S2.** Deming Regression of EDTA plasma and serum samples for OxPL-apoB assay.

**Figure S3.** Matrix correlation of EDTA plasma and serum samples for OxPL-apo(a) assay.

**Figure S4.** Deming Regression of EDTA plasma and serum samples for OxPL-apo(a) assay.
